# Nurse Leader Expertise for Pandemic Management: Highlighting the Essentials

**DOI:** 10.1093/milmed/usab066

**Published:** 2021-09-01

**Authors:** Susan G Hopkinson, Bonnie Mowinski Jennings

**Affiliations:** Center for Nursing Science and Clinical Inquiry, Tripler Army Medical Center, Honolulu, HI 96819, USA; Nell Hodgson Woodruff School of Nursing, Emory University, Atlanta, GA 30322, USA

## Abstract

The COVID-19 pandemic requires military nurse leaders in various patient care settings to engage in disaster response. Evidence supports essential leadership attributes for nurses that include skilled communication, organizational influence, and personnel management. Yet, nursing expertise that shapes nurse leader responsibilities during disaster management remains unclear. A description of how military nurse leaders contributed their nursing expertise during the COVID-19 pandemic response at one U.S. Military health care facility is provided to begin to delineate disaster management responsibilities.

## INTRODUCTION

Nurses’ integral role in disaster preparation and response is well-recognized worldwide.^1^ Nurses often feel ill-equipped, however, to respond during disasters.^2^ Moreover, little attention is given to preparing nurse leaders for their responsibilities during disasters.^3,4^ Nurse leaders, both military and civilian, play a particularly important role in influencing and facilitating the preparation and response of nursing personnel during disasters.^4^ Thus, looking at how nurses enacted leadership at one military treatment facility during the current COVID-19 pandemic response serves to highlight nursing expertise essential for future disaster response.

### Background

China first reported a cluster of pneumonia cases from an unknown cause on December 31, 2019.^5^ The pathogen was identified as a new coronavirus, SARS-CoV-2, that yielded the disease known as COVID-19. Within 2 weeks, the first case of COVID-19 outside China was reported. By the end of January 2020, there were 82 cases in 18 additional countries. A pandemic was declared on March 11. Sources of uncertainty surrounding COVID-19 included lack of clarity on its transmission mode, a potentially prolonged incubation, varied symptom presentation, and diagnostic testing inconsistencies.^6,7^

The uncertainties and rapid spread of COVID-19 present challenges for nurse leaders. Although it is recommended that nurse leaders take courses in incident management and incident command systems as part of disaster preparedness training, that knowledge is not enough.^4^ Nurse leaders must also exhibit essential leader skills and abilities such as problem solving, decision making, skilled communication, and ability to prioritize. Evidence supports that having positive leadership styles and traits is essential for the nurse to be an effective *leader* during disaster management.^3,4^

Although management and leadership are both important functions, they are different. Leadership is about relationships while management focuses on maintaining order, planning, organizing, and attending to rules and details. Despite these differences, in the U.S. Army, leadership and management are often merged and used as one concept.^8^ This overlap of terminology is also true for nursing.^9^ Because disaster management can be interpreted as part of leadership,^4,7^ the term nurse leader is used to describe nurses in either leadership or management positions.

Missing from the literature is a clear statement about the nursing expertise that a nurse leader brings to disaster management, and how that nursing expertise drives how the nurse leader uniquely contributes to the interdisciplinary team response. Nursing expertise encompasses the accumulated experiential knowledge that nurses as leaders have developed over time.^10^ This knowledge is developed based on the activities that nurses engage in that are distinctive to the nursing profession. The purpose of this article is to describe how military nurse leaders at one U.S. Military treatment facility applied their nursing expertise in response to the COVID-19 pandemic.

### The Military Treatment Facility

Tripler Army Medical Center (TAMC) is a tertiary military health care facility located on Oahu, the most populated of Hawaii’s islands. The only inpatient military facility in the state, TAMC, health care beneficiaries comprise about 170,000 of the 1.4 million people living in Hawaii.^11^ The beneficiaries include active duty military, retirees, and family members of active duty and retirees. Because there is no Veterans Affairs (VAs) inpatient facility on Oahu, TAMC also provides inpatient care to veterans when space is available. The primary VA outpatient clinics are co-located with TAMC.

## NURSING STAFF

The nursing staff at TAMC comprises registered nurses, licensed practical nurses, medical technicians, nursing assistants, and administrative personnel who are either active duty military (mostly Army personnel) or government civilians. The outpatient nursing staff is primarily government civilians, whereas the inpatient nursing staff is about 60% military and 40% civilians.

Similar to nurses working in non-military facilities, all nursing personnel working in military facilities participate in competency training to sustain clinical skills appropriate to their assigned care units. Remaining competent in clinical skills minimizes the additional preparatory training needed for disaster response or when military nurses are deployed to areas of combat.

Distinctive to military nursing personnel, including leaders, is a requirement to demonstrate physical fitness and soldier skills (e.g., weapons proficiency and survival know-how). These requirements help mitigate the risk of illness and/or injury through optimizing physical fitness and well-being as well as ensuring endurance during adverse conditions. Military personnel are also required to attend resiliency training to assist them in coping with stress and anxiety.

## NURSE LEADERS AT TAMC

At TAMC, the nurse leader at the highest organizational level is the Chief Nursing Officer (CNO). The CNO has administrative supervision of all nursing personnel. Before the pandemic, the CNO strategically aligned nurses in leadership positions within departments traditionally dominated by physicians (e.g., medicine, surgery, and quality improvement). Nurse leaders, therefore, collaborated with multiple departments on a daily basis.

Military nurses undergo leadership training throughout their careers, often alongside military leaders from professions outside of health care. All military leaders receive training in personnel management and operations planning. Additionally, the military rank of officer nurses often places them in a peer-level relationship with leaders from other disciplines such as physicians and health care administrators.

Military health care leaders face unique demands related to keeping active duty military, including health care personnel, ready to deploy to areas of conflict. For instance, some TAMC military nursing personnel have secondary assignments to mobile units located in other areas of the USA or in other countries. When health care needs are increased because of a disaster or military conflict, these military personnel are mobilized to serve where they are needed. The personnel remaining at TAMC adjust to safely cover needed nursing care. Especially for the inpatient nursing units, the nurse leaders maintain open communication with other leaders in the facility regarding current inpatient capabilities based on staff availability. At times, this might necessitate reducing the available inpatient beds.

### Emergency Preparedness

An ∼175 page appendix in the existing emergency preparedness plan was important in TAMC’s initial response to the COVID-19 pandemic. The appendix addressed establishing an emergency operations center (EOC) comprised of interdisciplinary experts knowledgeable in areas such as infection prevention and control (IPC), facility management, logistics, and health care administration. The appendix included detailed information about patient flow into the facility, access restrictions, patient/visitor education, and staff education regarding anticipated IPC measures specific to a respiratory infection. Mentioned in less detail were bed expansion parameters, internal patient flow, and just-in-time training.

Biannual exercises conducted at TAMC focus on mass casualty preparedness for a natural disaster. Fortuitously, an interdisciplinary exercise conducted in January 2020 was based on a highly infectious disease scenario. The exercise tested and provided feedback on IPC procedures for highly infectious patients as they entered the emergency department (ED) and moved through admission into adult critical care.

### The Initial Response

As outcomes from the January exercise were being evaluated, TAMC staff members found themselves focusing on the real-life scenario of COVID-19. The ED leadership, including nurses, knew they had to identify patients who potentially had COVID-19. Personnel safety for ED staff was emphasized, including training in the proper use of personal protective equipment (PPE) and attaining recommended supplies. Large tents were erected outside the ED to screen and test patients suspected of having COVID-19 before their entry into the patient waiting area. The initial ED preparation represented an interdisciplinary effort.

Based on organizational structure, the TAMC ED was part of the department of medicine, a department that also included medical primary and specialty care outpatient clinics. The nurse leader aligned with the director of the department of medicine had the authority and flexibility to move nursing personnel from the outpatient clinics to work in the COVID-19 screening tents and assist with staffing in the ED. The nurse leaders in the ED then assumed responsibility to ensure that the appropriate just-in-time competency training was completed with the nursing personnel as they moved into unfamiliar work environments.

The TAMC EOC was activated mid-March 2020 to provide a centralized communication hub. Non-essential health care appointments and procedures were cancelled. Visitors were restricted, with few exceptions. Only TAMC personnel and select outpatients were allowed into the facility via two controlled entries, one of which was the ED.

With the ED flow of patients established, the EOC personnel focused on expansion efforts for an expected surge in COVID-19 inpatients. Although the numbers of COVID-19 positive cases were not yet high in Hawaii, TAMC leaders had to respond to deadlines for expanding bed capacity driven by a combination of DoD guidance, state mandates, pandemic prediction models, and the presentation of patients to health care facilities throughout Hawaii.

### **Nurse Leader Areas of Expertise (See Fig. 1**)

The absence of detailed information in the emergency preparedness plan prompted the CNO to assign nurse leaders to spearhead-specific efforts that fell within the realm of nursing expertise. These efforts included collaborating inter-departmentally to develop a bed expansion plan, establishing the internal flow of patients suspected or confirmed for COVID-19, identifying nursing personnel to cross-train, implementing just-in-time training, and providing emotional support for the staff.

**FIGURE 1.**
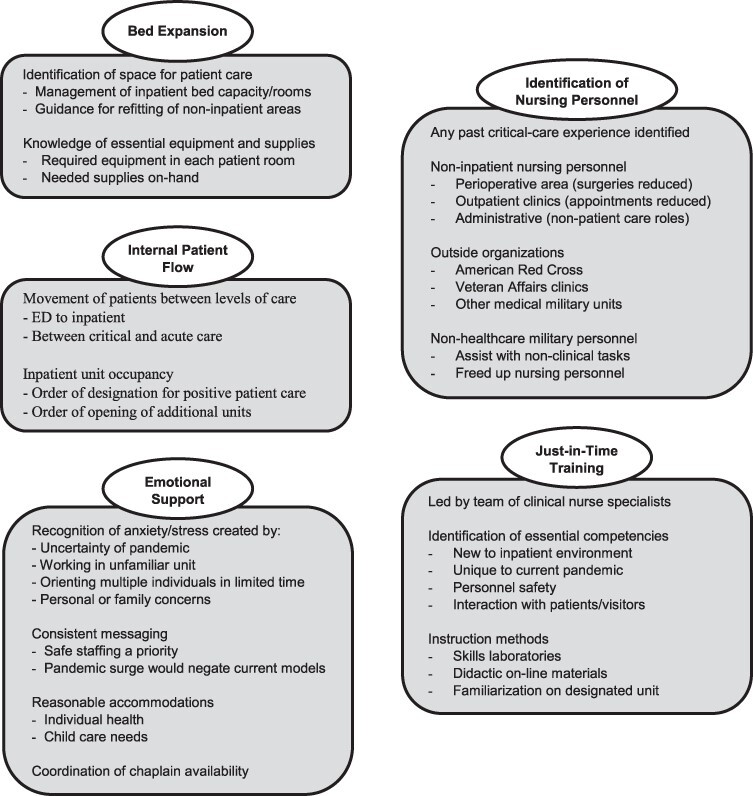
Contributions of nurse leader expertise to the pandemic response by topic area.

### Bed Expansion

Using national pandemic prediction models, TAMC inpatient beds needed to increase 3-fold. This required identifying space, refitting rooms for patient occupancy, procuring beds, and acquiring needed equipment and supplies. The nurse leaders were respected as experts regarding bed expansion requirements, with leaders from other departments such as facility management and logistics providing additional expertise and support. Interdisciplinary meetings always included nurse leaders to ensure all contingencies were identified. The nurse leaders guided the expansion based on their knowledge of the essential equipment and supplies needed to provide nursing care in the identified spaces. In just 1 month, TAMC achieved the 3-fold increase in projected bed demand within the existing physical structure by increasing capacity on existing units, refitting three non-inpatient areas into inpatient units, and reconfiguring space for a large open-bay unit.

### Internal Patient Flow

A focal point of patient flow concerned moving patients suspected of or positive for COVID-19 between levels of care, as well as designating restricted corridors and elevators to limit exposure. In collaboration with other departmental leaders, nurse leaders helped determine how these patients would move from the ED to inpatient units as well as between inpatient units. Contingency plans included the order in which additional beds and/or units would open and be designated for COVID-19 positive patients. The nursing knowledge of expected patient movement between units and support departments for procedures provided critical insight to establish a safe flow of inpatients.

### Identifying Nursing Personnel to Cross-Train

Inpatient units at TAMC have a core staff of nursing personnel who have demonstrated appropriate competencies based on the unit type. The bed expansion to accommodate projected numbers of patients with COVID-19, however, stressed the inpatient areas, especially the critical care and medical-surgical units. Simultaneously, military nursing personnel with secondary assignments to mobile units were being moved to areas where greater numbers of COVID-19 patients were anticipated. This left fewer competency trained nursing personnel on the inpatient units. Nurse leaders drew upon their expertise in personnel management, understanding of staffing models, and knowledge of patient care needs to expand and optimize nursing staff.

Through interdisciplinary collaboration with the non-nurse department directors, nursing personnel not working on inpatient units were identified to support existing inpatient staff. A priority was placed on identifying nurses with past critical care experience or those interested in supporting critical care. Requests for and identification of nursing personnel to work in the inpatient areas were guided by nurse leader understanding of safe staffing models and the possible need for contingency surge staffing.

Additional nursing staff were identified by reaching out through the EOC to other civilian and military organizations on Oahu. The American Red Cross identified 20 volunteers, over half of whom had a nursing background. The Chief Nurse of the co-located VA outpatient clinics also identified over 25 nursing personnel who volunteered to train for inpatient care roles. Other medical military units on Oahu that did not have inpatient beds also identified potential personnel to help with staffing. These medical units, however, had the concurrent challenge of juggling mission requirements to support areas outside of Oahu. The non-health care military units did, however, provide soldiers to assist with non-clinical tasks (e.g., gate guard and facility entry screener).

### Just-in-Time Training

Directed by a senior nurse leader, a team of clinical nurse specialists identified essential competencies for nurses who were assigned to new units, developed a training plan, and coordinated the just-in-time training for COVID-19 nursing care. The critical care physicians and nurse leaders developed additional, specialized training. The hospital education department provided logistical support for the training. During the month of April 2020, about 200 nursing personnel received just-in-time training—50 to support the critical care units and 150 to support the medical-surgical units. The just-in-time training included skills laboratories, didactic on-line materials and/or videos, computer documentation, and rotations to the inpatient units for hands-on experience.

The just-in-time training emphasized personal safety regarding PPE use. Another focus of the training was how to interact with patients who were isolated, unable to have visitors, and experiencing a disease about which little was known.^12^ A pamphlet with expected patient and family concerns was created and provided to all inpatient staff.

### Emotional Support for the Staff

Nurse leaders recognize that effective patient care is underpinned by staff who feel supported. Efforts to decrease anxiety and stress for the nursing personnel were integrated into the pandemic response. The uncertainties of the potential surge in patients, changes in roles, changes in routines, concerns for personal safety, and dealing with the different stressors for patients all tested the resilience of the nursing personnel.

Assigning nursing personnel to work in unfamiliar areas created anxiety. The nurse leaders conveyed a consistent message to reflect their understanding of the uncertainty and discomfort that accompanied working in an unfamiliar area.

Discomfort also was experienced by the nursing personnel on the inpatient units because of orienting multiple individuals in a short time. Concerns were raised regarding safe scope of practice and standards of care. The CNO clearly communicated that safe staffing was a priority, with the additional proviso that under pandemic surge conditions, current staffing models would no longer apply. Adapting standards of care is recognized as a necessary action under extreme circumstances such as pandemic care.^13^

Regardless of work area, some of the nursing personnel had personal or family member health concerns and others had childcare challenges. Such concerns were considered on an individual basis based on the belief that fair treatment did not mean treating everyone the same. Staff from the labor management and employee relations department was consulted frequently to determine appropriate actions. Chaplains were also available, as requested, to provide emotional support.

### The Continuing Response

The expected surge in COVID-19 patients did not occur on Oahu; thus, few COVID-19 positive patients were admitted to TAMC. Possible factors playing into this lower than expected number of patients testing positive included the geographical isolation of the island, the closure of the tourist and service industries, social isolation mandates, the sense of family within the Hawaiian culture, and adequate testing and contact tracing.^14^

Hawaii remains among the states with the fewest COVID-19 positive cases reported. As restrictions are lifted, it remains unknown to what extent the cases will surge. Nurse leaders at TAMC are, therefore, cognizant of continued stressors such as staff concerns about bringing the virus home to their families, having personal health issues, practicing in unfamiliar work environments, curtailing previous social plans, being unable to visit extended families, and delaying expected military moves to new assignments.

Even as the beds at TAMC are reduced to pre-COVID levels, the capability remains to quickly expand bed capacity. Identified nursing personnel are ready to supplement inpatient care. The patient flow would be re-established for individual patients suspected of or confirmed with COVID-19. Essential supplies, to include PPE, are being restocked with a plan for sustainment over an unknown timeframe. There is an opportunity to reexamine how to manage the impact of any pandemic as we enter a new reality of continued uncertainty.

## DISCUSSION

In the TAMC experience, it was evident that the military context assisted in the pandemic response, although even the military training and leadership experiences did not fully prepare the nurse leaders for dealing with the uncertainty of COVID-19. As noted by Veenema et al.,^1^ not only do nurses need disaster preparedness training and knowledge, nurse leaders must also hone disaster-related skills through practical exercises. The nurse leaders at TAMC capitalized on their nursing expertise to lead the efforts involving bed expansion, patient flow, identifying nursing personnel to cross-train, just-in-time training, and emotional support.

Perhaps because of how rank alters the power structure within the military, the nurse leaders at TAMC, all of whom were of similar rank to leaders from other disciplines, had key responsibilities during the response phase. Additionally, the collegial relationships among the interdisciplinary leaders at TAMC supported meeting demands quickly.

As COVID-19 persists, and with the possibility of another pandemic from a different etiology, nurse leaders must engage in their facility’s disaster planning. Particular attention should be given to clearly identifying the nurse leaders’ expertise in areas such as the implementation of just-in-time training. Although the exact focus of the training may not be known until the event happens, nurse leaders should identify who will develop and conduct the training. In the TAMC situation, the two focus areas were ensuring that nurses familiar with inpatient care received additional critical care training and the nursing personnel who did not routinely work in inpatient areas received familiarization training.

A prevalent concern noted in the literature is whether nursing personnel will be willing to work during a pandemic.^15^ Nurse leaders must recognize what factors influence willingness to participate in patient care during pandemics, such as perceptions of personal safety, previous training, confidence in one’s own skills and role, and knowledge of pandemic risk.^15^ Guidance for crisis standards of care for nursing from professional organizations may also be useful.^16^ Ensuring increased health risk concerns are addressed through reasonable accommodation processes can reduce inconsistencies amongst leaders and enhance the staff’s confidence in the nurse leaders’ decisions.

The resiliency and mental health of nursing personnel also need close monitoring. Part of the military culture is to take care of the team. Leaders and co-workers checked on each other, with liberal time given if needed (within legal parameters) to deal with childcare, personal, or other needs. Nurse leaders must continue to monitor the emotional state of the nursing personnel and provide needed support, especially as the circumstances surrounding the COVID-19 pandemic remain uncertain.

## CONCLUSION

Although it may be difficult to discern the exact expertise that nurse leaders contribute to disaster and/or pandemic management, the initial description in this article of applied expertise based on real-life experience provides a basis for discussion. Nurse leaders have developed this expertise over time based on their exposure to the domain of nursing that includes management of patient beds, patient flow, and competency training as well as emotional support of the staff as part of routine practice. Nurse leaders uniquely contribute this expertise for the functioning of the interdisciplinary team, regardless of the circumstances. Having nurse leaders integrated within all departments at TAMC assisted in maximizing upon their expertise and collaborative relationships to optimize the overall response to the pandemic.

## References

[1] Veenema TG , Griffin A, Gable AR, et al: Nurses as leaders in disaster preparedness and response—a call to action. J Nurs Scholarsh 2016; 48(2): 187–200. 26869230 10.1111/jnu.12198

[2] Labrague LJ , Hammad K, Gloe DS, et al: Disaster preparedness among nurses: a systematic review of literature. Int Nurs Rev 2018; 65(1): 41–53. 28295314 10.1111/inr.12369

[3] Knebel AR , Toomey L, Libby M: Nursing leadership in disaster preparedness and response. Annu Rev Nurs Res 2012; 30(1): 21–45. 24894051 10.1891/0739-6686.30.21

[4] Veenema TG , Deruggiero K, Losinski S, Barnett D: Hospital administration and nursing leadership in disasters: an exploratory study using concept mapping. Nurs Adm Q 2017; 41(2): 151–63. 28263273 10.1097/NAQ.0000000000000224

[5] World Health Organization : WHO timeline—COVID-19. Available at https://www.who.int/emergencies/diseases/novel-coronavirus-2019/interactive-timeline#event-0; accessed February 11, 2021.

[6] Harapan H , Itoh N, Yufika A, et al: Coronavirus disease 2019 (COVID-19): a literature review. J Infect Public Health 2020; 13(5): 667–73. 32340833 10.1016/j.jiph.2020.03.019 PMC7142680

[7] Singhal T : A review of coronavirus disease-2019 (COVID-19). Indian J Pediatr 2020; 87(4): 281–6. 32166607 10.1007/s12098-020-03263-6 PMC7090728

[8] Gallagher CR : Muddling leadership and management in the United States army. APOJ 2016; 108: 16–32.

[9] Jennings BM , Scalzi CC, Rodgers JD, Keane A: Differentiating nursing leadership and management competencies. Nurs Outlook 2007; 55(4): 169–75. 17678681 10.1016/j.outlook.2006.10.002

[10] Hutchinson M , Higson M, Cleary M, Jackson D: Nursing expertise: a course of ambiguity and evolution in a concept. Nurs Inq 2016; 23(4): 290–304. 27264695 10.1111/nin.12142

[11] World Population Review : Hawaii population 2020. Available at https://worldpopulationreview.com/states/hawaii-population/; accessed August 5, 2020.

[12] VITALtalk : Available at https://www.vitaltalk.org/; accessed August 5, 2020.

[13] American Nurses Association : Adapting standards of care under extreme circumstances: guidance for professionals during disasters, pandemics, and other extreme emergencies. 2008. Available at http://www.calhospitalprepare.org/sites/main/files/file-attachments/14__adaptingstandardsofcare.pdf; accessed January 27, 2021.

[14] Warner M : How Hawaii became a rare COVID success story. *Politico*, 2020. Available at https://www.politico.com/news/magazine/2020/06/19/hawaii-covid-success-story-322919; written June 19, 2020; accessed August 5, 2020.

[15] Aoyagi Y , Beck CR, Dingwall R, Nguyen-Van-Tam JS: Healthcare workers’ willingness to work during an influenza pandemic: a systematic review and meta-analysis. Influenza Other Respir Viruses 2015; 9(3): 120–30. 25807865 10.1111/irv.12310 PMC4415696

[16] U.S. Department of Health and Human Services : Topic collection: crisis standard of care. Available at https://asprtracie.hhs.gov/technical-resources/63/crisis-standards-of-care/0; accessed August 5, 2020.

